# How personalisation programs can exacerbate socio-economic inequities: findings from budget utilisation in the Australian National Disability Insurance Scheme

**DOI:** 10.1186/s12889-022-13301-x

**Published:** 2022-05-03

**Authors:** Eleanor Malbon, Megan Weier, Gemma Carey, Thomas Writer

**Affiliations:** Centre for Social Impact, University of New South Wales, Sydney, NSW 2052 Australia

**Keywords:** Personalisation, Social policy, Equity, Individual budgets, Social gradient

## Abstract

**Background:**

Researchers and policymakers are increasingly concerned that personalisation schemes in social and health care might be worsening social and health inequities. This has been found internationally, where better outcomes from such schemes have been found amongst those who have higher education and more household income.

**Method:**

This study looks at one of the world’s largest personalisation schemes, the Australian National Disability Insurance Scheme. Using publicly available data we examine the allocation and utilisation of NDIS funds according to social gradient.

**Results:**

We find that the rate at which people with disability ‘spend’ or effectively use their disability care funds follows a social gradient. That is, those in areas of higher socioeconomic disadvantage are not spending as much of their allocated budgets on care services across the year compared to people in areas of higher socioeconomic advantage. This represents a clear issue of equity in the use of public money to people with disability in Australia.

**Conclusion:**

We argue that this points to the need to provide targeted supports for the use of disability care funds in areas of higher socioeconomic disadvantage. Without effective supports for fund use, the NDIS and other personalisation schemes may be positioned to worsen existing social inequalities.

## Introduction

There is a long running concern, amongst policy makers and academics alike, regarding whether health and social inequities are ameliorated or exacerbated by different forms of social welfare policies [1, 2]. The impact of national welfare policy on health and social inequities has been analysed extensively, in areas ranging from employment to education [2, 3]. Even where the same services are offered ‘universally’ (where everyone received the same service), their uptake can be unequal [4]. This was famously highlighted in Hart’s [5] inverse care law, which holds that the availability of good medical care tends to be least available in the areas where it is most needed, and most available in areas where it is least needed.

As new forms of policy design emerge, these concerns persist [6]. One of the latest global trends in social and health policy are ‘personalisation approaches’ [7]. Personalisation programs are aimed at providing tailored services to citizens based on their particular service needs. Personalisation programs can be targeted to a variety of needs, such as young people in the education sector or people with disability. They do this through the use of personal budgets or vouchers, with which individuals can purchase services from a service market thereby increasing choice and control [8]. While personalisation programs are most often aimed at giving users more choice and control over the services they use questions have been raised about their effectiveness. For example, Flemming [9] found that outcomes of personalisation schemes are variable and not necessarily better than other forms of policy delivery. Meanwhile, emerging research has found that personalisation may in fact create inequities, and exacerbate existing ones [6, 10]. While improved equity outcomes may not be a specified goal of personalisation programs, by virtue of their status of welfare program it has been argued that they ought to improve or at least not worsen equity outcomes [6, 10].

This paper looks more closely at the question of personalisation and inequality, examining national data from the Australian National Disability Insurance Scheme (NDIS) – one of the largest personalisation programs in the world. We analyse publicly available data about the size of funds and the ability of citizens to utilise these funds by service area relative to socioeconomic advantage and disadvantage; conceptualised using the Index of Relative Socio-Economic Advantage and Disadvantage (IRSAD) which is used as a ranking system for geographic socio-economic status in Australia. This is the best available proxy for the socio-economic disadvantage of NDIS participants (as no household income data or the like is collected or released by government). Our findings indicate the degree to which participants utilise their welfare funds differs according to socio-economic disadvantage and advantage. Our findings suggest that those in areas with higher levels of socioeconomic disadvantage are less able to use their individual budgets to purchase the services they need. This points to the need to provide targeted supports for fund implementation in low socio-economic areas. Without effective supports for fund implementation, the NDIS is positioned to worsen existing social inequalities.

## Background

### Personalisation programs and equity

Personalisation programs aim to enable citizens to gain highly tailored services specific to their particular service needs [11, 12]. Within broader debates about the welfare state and its structure, personalisation can be understood as a form of ‘particularist’ approaches to social policy and health care. Particularism aims to address differences between individuals on the basis of diversity of needs, moral frameworks and social expectations. Particularism requires an appreciation of the different social identities of different groups (requiring investigation of values, wants, norms and needs). Particularist principles are said to allow for, and encourage, empowerment and a diversity of supply (e.g. heterogeneous services which take account of cultural and ethnic identities), thereby better catering to different groups and improving inequalities [4].

While personalisation programs have common aims, such as enabling citizens in personal goal setting and enabling choice and control, there are differences in the design and delivery of specific systems. Dickinson [8] highlights differences in personalisation models, including variation in:

• what individuals are allowed to spend money on;
• who manages this money/resources;
• levels of scrutiny over its use;
• the mechanisms through which choice and control are operationalised (e.g. service markets or other arrangements)

The most common mechanisms and administrative arrangement for enabling or operationalizing personalization is market structures, whereby participants purchase services from ‘market-like’ arrangements [12].

How personalisation schemes are designed and administered has implications for the experiences of different social groups using them and, in turn, inequality [6]. The design, governance and administrative processes within a personalisation program influences the level and type of administrative and negotiation skills required to successfully engage in and benefit from personalisation schemes [13–16]. In the UK, research has shown that people are more likely to benefit from personalisation if they are employed, have surplus financial resources, are educated and have strong social networks [7]. In Australia, research indicates that people with higher incomes and education levels are more likely to get their support needs met [10, 17]. A recent meta-review of international evidence shows that experiences of personalisation are positively associated with a range of factors that can be considered proxies for socio-economic position, including income, education and bridging social capital [6]. Flemming et al. [9] conducted a systematic review of personalization programs worldwide, and found that they are administratively complex in nature and often difficult for citizens to negotiate as a result.

Within social welfare debates there is growing attention on the potential for personalisation schemes to benefit higher socio-economic groups more than lower socio-economic group [6, 10]. Malbon et al.[10] found that active control over spending NDIS funds (known as ‘self management’), access to robust disability service markets and bureaucratic accessibility all contribute to how a citizen derives benefit from the NDIS. In a review of research on personalisation schemes and inequities, Carey et al. [6] observed that no studies explicitly examined the outcomes of individuals participating in personalisation schemes by socioeconomic status. However, they highlight that a number of factors have been identified as important for serving benefit from personalised schemes including levels of education, being employed, having capable networks, household income, skills in navigating complex systems and the capacity to self-manage budgets [6].

Further, Carey et al. (2019) has emphasised the impact of class on the design and implementation of personalisation schemes, noting that the increased advocacy required to navigate highly complex administrative schemes developed by middle class bureaucrats means that very principles that underpin personalisation schemes can leave particular social groups vulnerable, while “privileging users who have the best capacity to navigate the system” who are often from higher socio-economic backgrounds (page 8).

### The National Disability Insurance Scheme

The National Disability Insurance Scheme (NDIS) is a personalisation program for the support of people with significant and permanent disability in Australia [18]. The NDIS currently supports around 400,000 people, 150,000 of whom are receiving disability support for the first time [19]. First proposed in 2010, the program passed through a series of trial stages before beginning national implementation in 2017 [20]. The NDIS uses the mechanism of individual budgets to allocate funds to people with disability according to their disability related needs and goals. These NDIS budgets are then available to be spent by the person with disability or their agents in the disability care market, according to rules set by the NDIS.

There are two aspects to individual NDIS funding (also known as ‘plans’). There is the process for determining the amount of funding that a person with disability will be allocated to spend on their care to meet their self-defined goals (known as the ‘planning meeting’). Following the determination of funds, there is the process of spending or utilising the funds in which a person with disability or their agents contract a set of service providers using the NDIS funds (known as ‘plan implementation’).

Early concern about equity in the NDIS related to whether people of the same disability were receiving similar levels of NDIS funding [17]. Later concerns revolve around whether and how NDIS funds are spent on care  [10], with Carey et al. [6] contending the theory that NDIS benefit may be related to class status.

The National Disability Insurance Agency (NDIA) has concluded that higher fund spend (titled ‘plan utilisation’) is associated with better health and social outcomes for NDIS participants in both childhood and as adults. Because prices are standardised in the NDIS, a greater rate of fund spend translates to a greater amount of services accessed. For those in early childhood, the NDIA [21] conclude that “Higher baseline plan [fund] utilisation is a strong predictor of a positive response across all five areas surveyed.” (p6). Similarly, for adults they conclude that “Higher baseline plan [fund] utilisation is a strong predictor of a positive response across all eight [service] domains.” (p6).

To better define this issue, we sought to establish whether NDIS data shows difference in both NDIS funding allocated and NDIS funding spent according to geographical socio-economic status. Based on limited work exploring socioeconomic status and NDIS funding, this study focuses on two exploratory hypotheses:

• Hypothesis 1: Higher average approved plan budgets would be associated with higher levels of socioeconomic advantage.
• Hypothesis 2: Higher average rates of plan utilisation would be associated with higher levels of socioeconomic advantage.
• Hypothesis 2a: This relationship would remain significant once average approved plan budget is controlled for.
• Hypothesis 2b: This relationship will not significantly vary across disability support class types.

The analysis uses data currently provided by the National Disability Agency (NDIA), covering approved plan budgets and rate of utilisation, reported as averages for each NDIA service district. The hypothesis and dependent variables are found in Table 1.

**Table 1:** Dependent and independent variables identified in analyses

	Independent variable(s)	Dependent variable(s)
Hypothesis 1	Average ISRAD score for service district	Average approved annual plan budget
Hypothesis 2	Average ISRAD score for service district	Average rate of utilisation
Hypothesis 2a	Average ISRAD score for service district Average approved annual plan budget	Average rate of utilisation
Hypothesis 2b	Average ISRAD score for service district	Average rate of utilisation – core activities Average rate of utilisation – capacity building activities Average rate of utilisation – capital activities Average rate of utilisation – core activities (including SILSDA) Average rate of utilisation – capacity building activities (including SILSDA) Average rate of utilisation – capital activities (including SILSDA)

## Methods

### Data sources

#### Relative Socio-Economic Advantage and Disadvantage

The most common and robust indices of Australian socio-economic disadvantage and advantage are calculated every five years using the Australian Census. The Australian Bureau of Statistics produces four indexes each census cycle, known as Socio-Economic Indexes for Areas (SEIFA), and includes four indices that reflect different aspects of relative advantage and disadvantage (e.g. disadvantage only, education and occupation, or economic resources). This study used the 2016 Index of Relative Socio-economic Advantage and Disadvantage (IRSAD), which summarises the economic and social conditions for individuals and households within particular areas [22]. This particular Index reflects not only a lack of advantage (represented by a low score) but also areas of relative greater general advantage (represented by high scores). The Index is constructed by the Australian Bureau of Statistics, and is comprised of variables including proportion of households with low income, proportion of houses with no internet connection, and the proportion of people with long-term health conditions or disability and need assistance with core activities. The IRSAD is reported against Local Government Areas (LGAs), an approximation of gazetted local government boundaries as defined by individual Australian States and Territories. There are 562 LGAs in Australia.

#### NDIS participant funds and utilisation

The NDIA provides quarterly data updates on participant numbers and average support budgets, split geographically by LGA and NDIA service district. There are 86 NDIA service districts that group together multiple LGAs; mapping against 2016 geographic boundaries is made available by the NDIA and was used for this analysis [23]. The latest data, reported in June 2021, and data from the year prior (June 2020) was used for this analysis.

This dataset represents the only regularly updated administrative information for NDIS participant numbers and budgets. ‘Average support budget’ refers to the annual approved budget averaged across all participants within the service district. No information is currently provided on the range or median approved amount. Outliers in approved budgets that influence the average reported budget for a service district are not reported; this aggregated form of data is the best currently available data on the NDIS, and any service districts with significantly higher or lower average approved budget amounts were not removed from the analysis in order to understand how equitable the scheme currently is based on publicly shared data.

Separately, the NDIA also provides quarterly updates of average fund utilisation reported across State/Territory (reported as a proportion), service district, support class (4 categories—capacity building, capital, or core, and ‘all’), disability group (16 categories—15 NDIS identified groups, and ‘all’), supported independent living (SIL) or supported disability accommodation (SDA) (3 categories – yes, no, and ‘all’), and age band (10 categories—0 to 6, 7 to 14, 15 to 18, 19 to 24, 25 to 34, 35 to 44, 45 to 54, 55 to 64, 65 + , and ‘all’). For the purpose of this analysis, ‘all’ categories were used for age. Further, unless noted, utilisation rates for participants receiving supported independent living or in supported disability accommodation were excluded, as it was assumed that the majority of budgets would be used for participants requiring essential assisted living care. The latest data, reported in June 2021, and data from the year prior (June 2020) was used for this analysis.

#### Data aggregation

Participant utilisation is only provided at the NDIA service district level so could not be compared directly to LGA IRSAD scores. Average IRSAD scores were calculated by aggregating the IRSAD score for all LGAs within each service district area. Data were aggregated using Pivot Tables and VLOOKUP functions in Microsoft Excel. Average participant budgets and reported fund utilisation rates for June 2020 and June 2021 were aggregated into one dataset for analysis. The NDIA provides data for fund budgets for ‘Other Unincorporated Territories’ (also known as remote offshore territories). Rates of plan utilisation (representing 24 participants) are not reported for these territories, and were excluded from analysis.

#### Analysis approach

Descriptive analysis was undertaken of average individual fund amounts and rates of utilisation for each State and Territory. Relationships between relative advantage or disadvantage and NDIS budgets and fund utilisation were analysed using linear regression modelling. The independent variable was the average IRSAD score for each NDIA service district. Pearson’s correlations were used to explore the strength of relationship between the various disability types and the utilisation of individual funds. All analysis was conducted in SPSS Statistics v.25.

## Results

Table 2 summarises State and Territory IRSAD scores, alongside average approved budgets and average rates of utilisation in June 2020 and June 2021. IRSAD scores ranged between 697 and 1131, with higher scores indicating higher levels of relative advantage. In 2020, the national average approved NDIS budget was $75,047 (SD $29,932), and an average only 57.14% of budgets were being used (SD 8.70). Average budgets decreased slightly in 2021 ($73,538, SD $21,399), and average utilisation slightly increased (61.87%, SD 6.22). The Northern Territory had scores indicating the greatest disadvantage across the country, as well as the highest average approved NDIS budget. The Northern Territory has the highest proportion of people living in remote and very remote communities, as well as the highest proportion of Aboriginal and Torres Strait Islander residents – both factors associated with greater need for health and social supports. The ability to access appropriate NDIS services appear to be lacking in the Northern Territory – the average utilisation rate across all disabilities for the Territory was 42% in 2020; increasing to 54% in 2021. In contrast, the Australian Capital Territory (ACT) is the smallest Territory within the country and had the highest average scores of relative advantage across the country. It also had substantially higher average rates of utilisation across all disabilities (66% in 2020, 68% in 2021), while also having a lower average approved budget of $62,000, compared to the national average of $75,047.62. However, the average approved budget for ACT service area jumped substantially in 2021, doubling to $124,000. The ACT is counted as one NDIS service area, whereas other States and Territories include at least 4 service areas where budgets are averaged across, however this substantial increase in approved funding is worth noting and seeking further investigation if addressing socioeconomic inequity is a concern for the NDIS program.

**Table 2:** Average index scores for relative disadvantage, approved NDIS budget, and rate of plan utilisation, by State and Territory

State/Territory *(N NDIA service districts)*	Average ISRAD	Average Approved NDIS Budget ($)		Average rate of plan utilisation (%)	
		**June 2020**	**June 2021**	**June 2020**	**June 2021**
**All *(84)***	**964.37 (78.02)**	**75,047.62 (29,932.42)**	**73,537.50 (21,399.72)**	**57.14 (8.70)**	**61.86 (6.22)**
Australian Capital Territory *(1)*	1,089.00	62,000	124,000	66.00	68.00
New South Wales *(16)*	990.92 (62.75)	71,312.50 (6,838.31)	70,066.67	63.00 (6.60)	66.80 (5.17)
Northern Territory *(7)*	842.37 (135.27)	136,857.14 (48,450.76)	125,666.67 (45,548.51)	42.57 (10.29)	54.00 (7.51)
Queensland *(13)*	942.45 (71.30)	74,000 (11,165.42)	72,307.69 (6,128.96)	60.92 (2.90)	65.31 (3.30)
South Australia *(12)*	964.55 (53.15)	67,416.67 (9,287.90)	67,000.00 (9,448.43)	54.92 (8.59)	58.67 (6.27)
Tasmania *(4)*	929.55 (32.15)	83,000.00 (10,360.18)	78,750.00 (8,995.37)	59.00 (8.16)	62.75 (1.26)
Victoria *(18)*	987.68 (45.19)	58,000.00 (16,215.46)	62,705.88 (6,668.63)	59.11 (4.92)	61.11 (2.83)
Western Australia *(12)*	990.20 (65.08)	67,500.00 (23,380.26)	69,083.33 (10,121.61)	51.75 (8.86)	59.33 (7.16)

Standard deviations are shown in parentheses. Based on June, 2020 data

Hypothesis 1: Higher average approved plan budgets would be associated with higher levels of socioeconomic advantage.

**Fig. 1:**
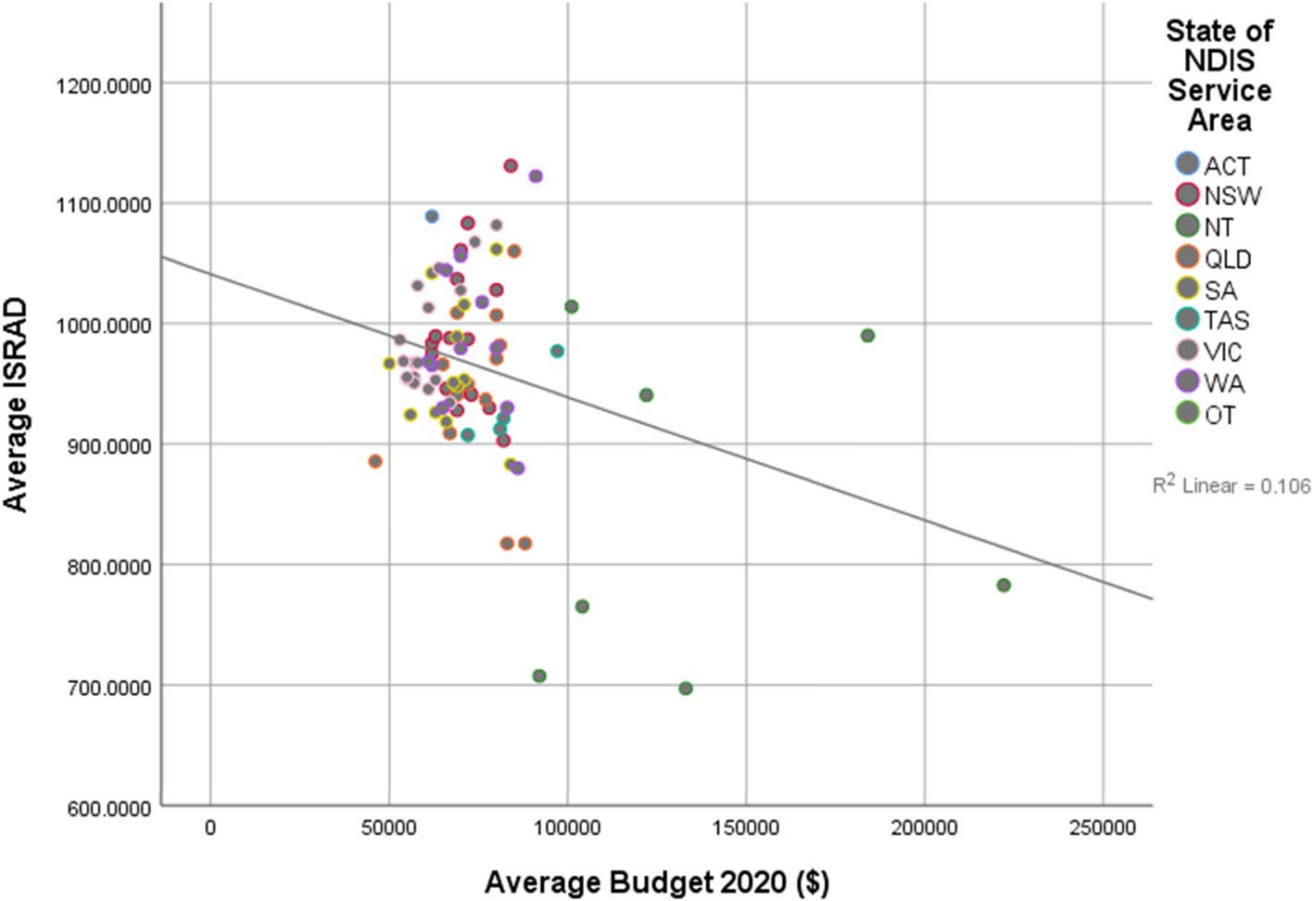
Relationship between relative disadvantage and average approved budget, June 2020

**Fig. 2:**
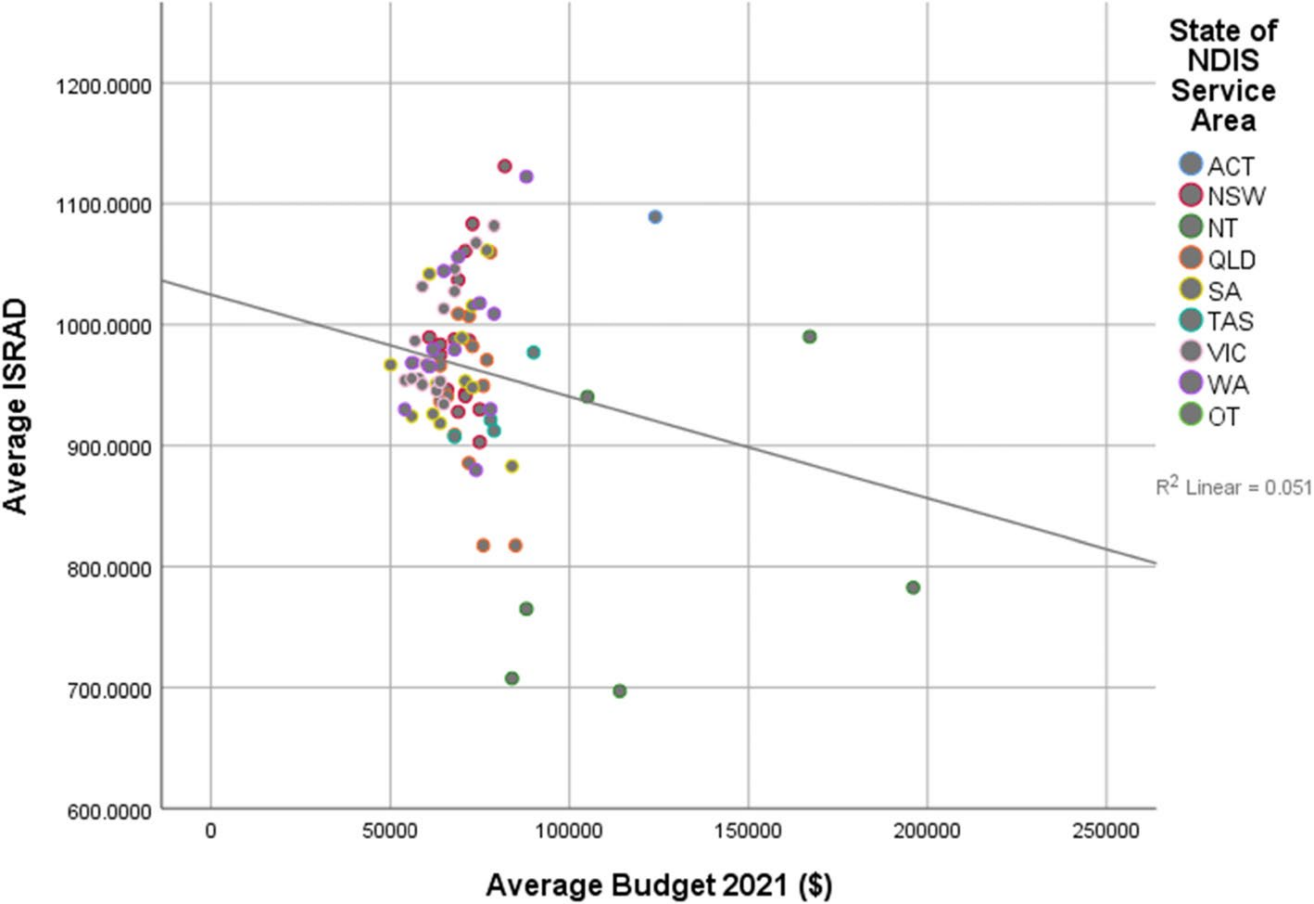
Relationship between relative disadvantage and average approved budget, June 2021

Linear regression results are shown in Table 3. Linear regression showed a significant negative relationship between IRSAD scores and approved budgets, whereby higher IRSAD scores, or higher levels of relative advantage, predicted lower average approved budgets in both 2020 (β = -0.325, *p* < 0.001, R^2 =^ 0.106) and 2021 (β = -0.227, *p* = 0.043, R^2^ = 0.051) (see Figs. 1 and 2, Table 3). While most average plan budgets clustered between $50,000 and $100,000, a number of higher approved budgets were outliers, particularly in service districts with higher levels of socioeconomic disadvantage.

**Table 3:** Linear regression of average index scores for relative disadvantage predicting averaged approved budgets and averaged rates of utilisation

	Average approved NDIS budget						Average utilisation rate					
	*June 2020*			*June 2021*			*June 2020*			*June 2021*		
	*B*	*SE B*	*β*	*B*	*SE B*	*β*	*B*	*SE B*	*β*	*B*	*SE B*	*β*
Constant	175,027.94	32,685.29		132,371.63	28,704.00		.002	.101		.204	.072	
Average IRSAD	-103.23	33.80	-.325**	-61.10	29.71	-.227*	.001	.000	.530***	.000	.000	.550***
R^2^	.106			.051			.281			.302		

^*^ *p* < .05, ** *p* < .01, *** *p* < .001

Hypothesis 2: Higher average rates of plan utilisation would be associated with higher levels of socioeconomic advantage.

**Fig. 3:**
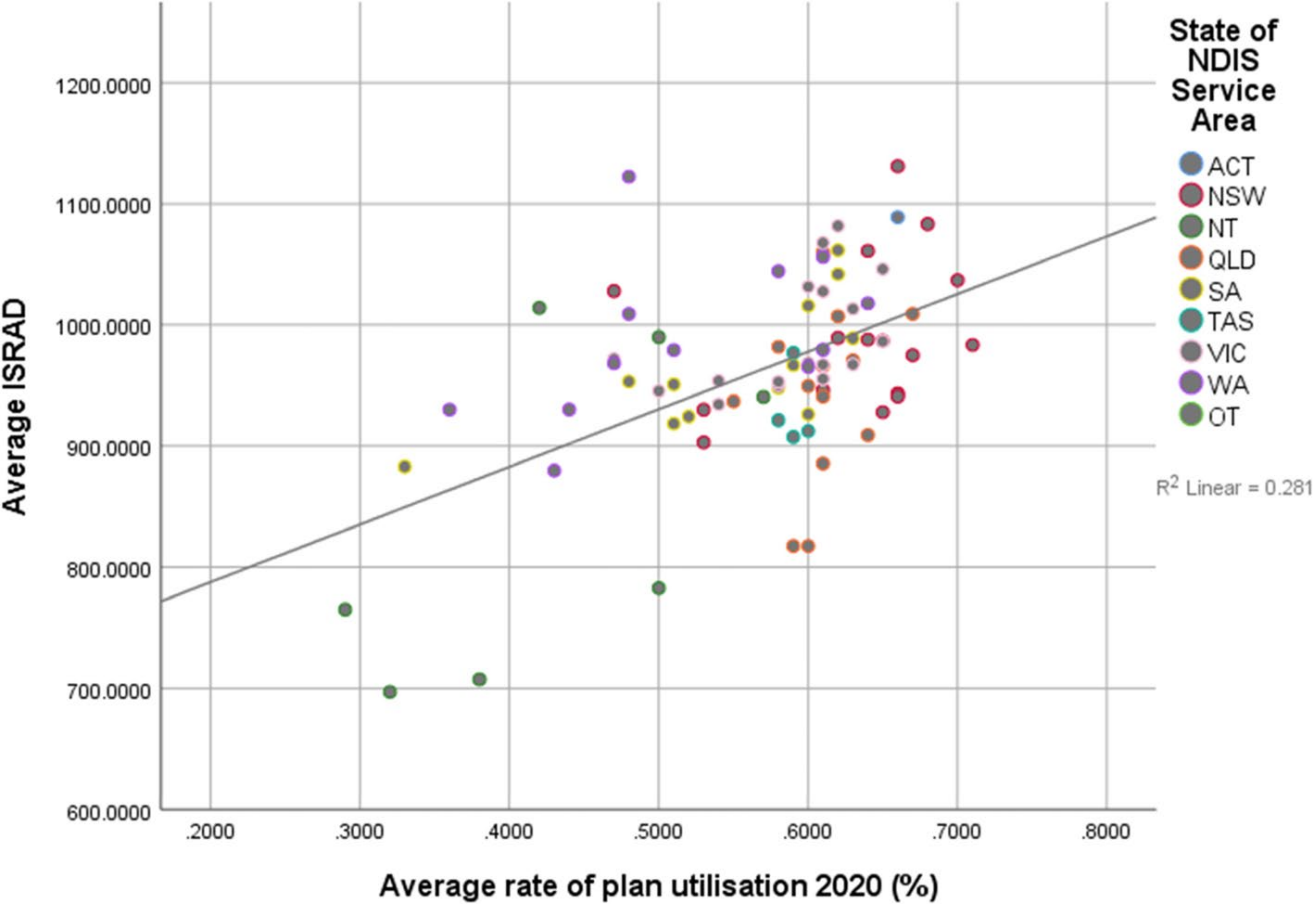
Relationship between relative disadvantage and utilisation rate of approved budgets (all disability types), June 2020

**Fig. 4:**
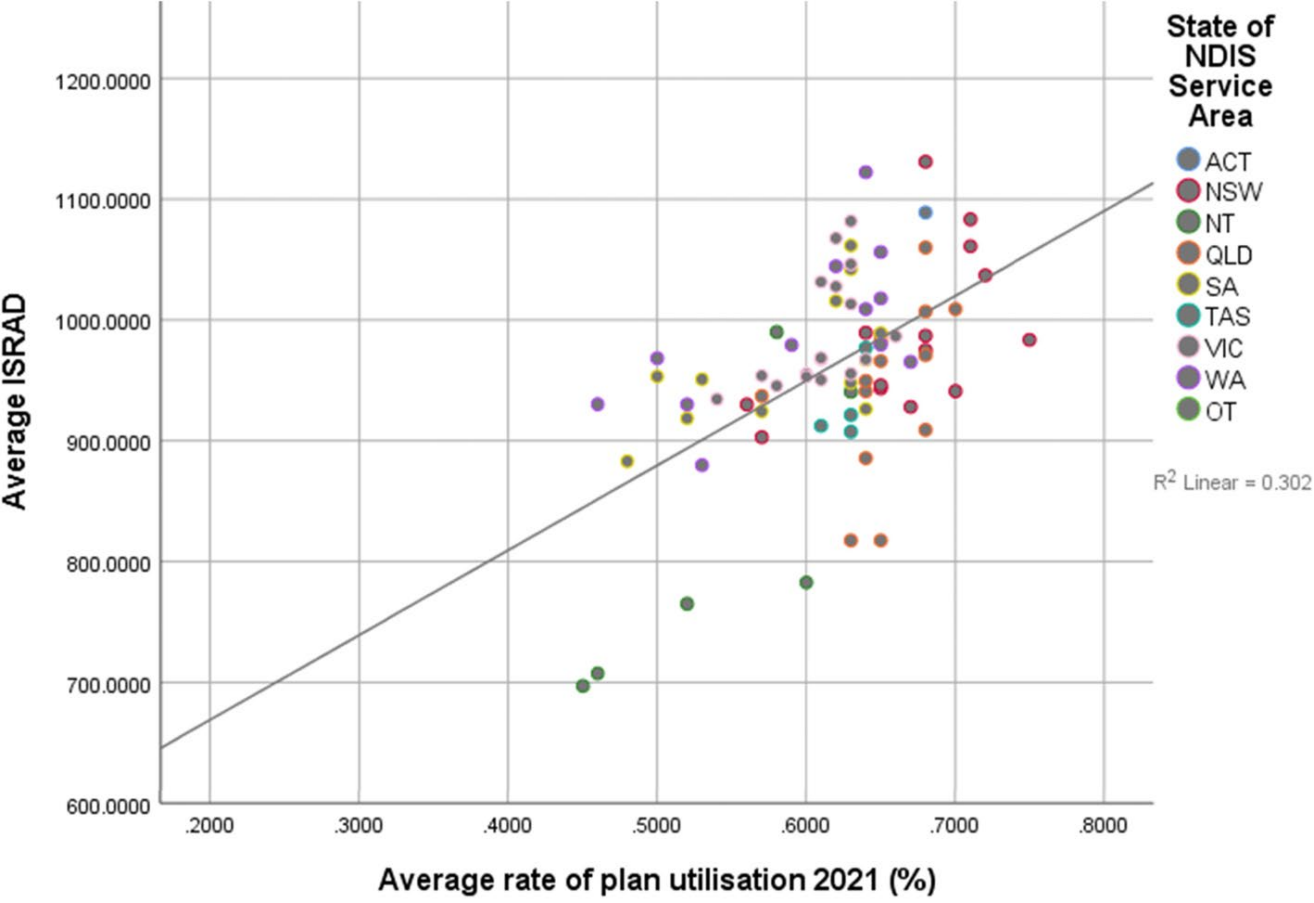
Relationship between relative disadvantage and utilisation rate of approved budgets (all disability types), June 2021

However, when looking at the relationship between relative advantage and disadvantage and fund utilisation rate, a significantly different relationship was observed (see Figs. 3 and 4). Higher levels of advantage (β = 0.530, *p* < 0.001, R^2^ = 0.281) predicted higher average rates of utilisation for ‘all’ disability types (excluding SIL and SDA clients), explaining 28% of model variance in 2020 (see Table 3). Similarly, higher levels of socioeconomic advantage (β = 0.550, *p* < 0.001, R^2^ = 0.302) predicted higher average rates of plan utilisation in 2021. That is, for an area such as the ACT, with the highest average IRSAD in the country (1089), clients with NDIS fund are also, on average, using a greater proportion of their allotted budget (66% in 2020, 68% in 2021). In contrast, a state such as Tasmania, which has an average IRSAD score (929.55) compared to the national average, predicts that clients on average utilise less of their budgets (59% in 2020, 62.75% in 2021). As Figs. 3 and 4 show, the relationship between socioeconomic advantage and higher rates of plan utilisation showed a clearer linear relationship, where participants living in areas of higher socioeconomic disadvantage were utilising a lower proportion of their approved plans. These two analyses indicate that while clients living in areas of greater socioeconomic disadvantage are being approved for higher individual funds, they utilise a smaller proportion of their funds compared to clients living in more advantaged areas.

Hypothesis 2a: Higher rates of plan utilisation and higher levels of socioeconomic advantage would remain significant once average approved plan budget is controlled for.

When predicting utilisation rates that excluded SIL and SDA (SILSDA), the average approved budget amount had a significantly negative contribution to the model above what was already predicted by average IRSAD scores in 2020 (β = -0.221, *p* = 0.025). This relationship was not significant in 2021 (β = 0.026, *p* = 0.792, see Table 4). This suggests that the approved budget amount may be contributing significantly to predicting the degree of fund utilisation across all disability types, for services that are not focused on supported independent living or disability accommodation. However, the relationship is not consistent across the two years of data and requires further investigation.

**Table 4:** Summary of Multiple Regression Analysis for Variables Predicting Plan Utilisation (All Disability types) (*N* = 84)

	Average utilisation rate—Exclude SILSDA						Average utilisation rate—Include SILSDA					
	*June 2020*			*June 2021*			*June 2020*			*June 2021*		
	*B*	*SE B*	*β*	*B*	*SE B*	*β*	*B*	*SE B*	*β*	*B*	*SE B*	*β*
Step 1												
Constant	-.007	.100		.204	.072		.183	.116		.316	.080	
Average IRSAD	.001	.000	.548***	.000	.000	.550***	.000	.000	.416***	.000	.000	.464***
R^2^	.292			.302			.173			.215		
Step 2												
Constant	.127	.114		.194	.081		.186	.136		.222	.088	
Average IRSAD	.001	.000	.476***	.000	.000	.556***	.001	.000	.414***	.000	.000	.517***
Average Approved NDIS Budget	.000	.000	-.221*	.000	.000	.026	.000	.000	-.005, ns	.000	.000	.231*
R^2^	.327			.303			.173			.266		
R^2^ Change	.044*			.001, ns			.000, ns			.051*		

^*^ *p* < .05, ** *p* < .01, *** *p* < .001

When looking at average fund utilisation rate that includes clients using funding for SILSDA, the average score for socioeconomic advantage was still a significant predictor, such that living in more advantaged areas predicted higher rates of fund utilisation in 2020 (β = 0.416, *p* < 0.001), however explained less model variance (17.3%) compared to predicting utilisation when SILSDA clients were excluded. This relationship between socioeconomic advantage and fund utilisation was stronger in 2021 (β = 0.464, *p* < 0.001), explaining 21.5% of model variance. This suggests that socioeconomic advantage may be less of an explanatory factor for utilisation for clients who are receiving support through SILSDA, however this relationship is still significant. Average approved budget amount did not significantly predict utilisation rates in 2020 (β = -0.005, *p* = 0.963), nor did it significantly contribute to the amount of variance accounted for by the regression model. In 2021, average approved budget amount explained additional variance for fund utilisation over and above socioeconomic advantage (β = 0.231, *p* = 0.024). This provides initial evidence that in 2021, having a higher approved budget may contribute to higher levels of utilisation. However, this inconsistent relationship requires further exploration rather than implying there is a consistent trend for higher approved budgets being related to higher rates of utilisation over and above level of socioeconomic disadvantage.

Hypothesis 2b: The relationship between rates of plan utilisation and higher levels of socioeconomic advantage would not significantly vary across disability support class types.

Linear regressions, modelling the relationship between average utilisation rate and average ISRAD scores for service districts, were conducted across three disability support class types – core, capacity building, and capital-, and were calculated when utilisation included SILSDA plans. In both June 2020 and June 2021 data, there was a significant positive relationship between utilisation rates and higher levels of socioeconomic advantage (see Table 5). All disability support class types were significantly associated with socioeconomic advantage, except for capital support in June 2020. Beta values were consistently high for capacity building activities (β ranging from 0.550 in June 2021 excluding SILSDA plans, to 0.592 when SILSDA plans were included), compared to core and capital support activities.

**Table 5:** Linear regression of average index scores for relative disadvantage predicting averaged utilisation rate by support class (*N* = 84)

	Average utilisation rate—Exclude SILSDA						Average utilisation rate—Include SILSDA					
	*June 2020*			*June 2021*			*June 2020*			*June 2021*		
	*B*	*SE B*	*β*	*B*	*SE B*	*β*	*B*	*SE B*	*β*	*B*	*SE B*	*β*
Core												
Constant	.027	.142		.065	.121		.253	.160		.421	.082	
Average IRSAD	.001	.000	.409***	.000	.000	.370**	.000	.000	.291*	.000	.000	.411***
R^2^	.167			.137			.084			.169		
Capacity Building												
Constant	-.081	.097		.204	.072		-.057	.099		.043	.075	
Average IRSAD	.001	.000	.555***	.000	.000	.550***	.001	.000	.535***	.001	.000	.592***
R^2^	.308			.302			.286			.351		
Capital												
Constant	.198	.289		.019	.074		.182	.283		.073	.134	
Average IRSAD	.000	.000	.147	.001	.000	.614***	.000	.000	.149	.000	.000	.337**
R^2^	.022			.377			.022			.113		

^*^ *p* < .05, ** *p* < .01, *** *p* < .001

## Discussion

This study sought to understand how socio-economic position may affect individual fund allocation and utilisation in the NDIS, as a means by which to investigate both specific issues within the NDIS regarding equity as well as broader emerging questions about personalisation schemes and inequality. Crucially, we found that people who live in areas of higher socioeconomic advantage are more likely to utilise more of their approved NDIS budgets. We conclude that the NDIA must look beyond equality in the allocation of NDIS funds, to ensuring that people in lower socioeconomic areas are given additional support to utilise their allocated funds.

As this study is an initial investigation into socioeconomic disadvantage and its relationship with personalisation schemes and equity, there were some exploratory hypotheses that guided the analysis. Firstly, it was hypothesised that there would be a positive relationship between approved plan budget value and socioeconomic advantage. In both 2020 and 2021 data, the inverse of this relationship was demonstrated. There was a negative relationship where higher average budgets were reported in service districts that higher levels of socioeconomic disadvantage. As the current data that is available is reporting on averages, it is possible that a small number of outliers with larger approved budgets are contributing to this higher average. Possible explanations for this are that there are less people with disability in these more economically advantaged areas, or people with disability with less high needs or less people with a disability significant enough to quality for the NDIS living in these areas. Information is not currently available to better understand the underlying drivers for the findings presented in Figs. 1 and 2. However at the planning stage, it appears that the NDIS administrative system is managing to uphold equity principles in the approving and allocation of budgets.

The second hypothesis was concerned with the degree to which clients would be able to access support, measured using the average reported utilisation rate for each service district. In both 2020 and 2021, there was a significant positive relationship between utilisation rate and higher levels of socioeconomic advantage. This relationship remained significant when including utilisation rates for plans that included supported independent living and disability accommodation. In June 2020 data, adding the average approved budget significantly contributed to the regression model when understanding the utilisation rates excluding SILSDA, while the average approved budget significantly added to the regression model when analysing utilisation rates which included SILSDA plans in June 2021. That is, higher average budgets also are related to higher utilisation rates, over and above higher levels of socioeconomic advantage. However this relationship was inconsistent so can only be an initial observation that could be monitored over time.

Different disability support class types indicate the type of care supports that is available to participants. While core and capacity building activities may require ongoing engagement with services (such as appointments, transport and therapies), capital activities are more likely to be single purchases such as buying equipment or getting home adjustments made. In June 2020, there was no significant relationship between socioeconomic advantage and utilisation rates of capital plan budgets. Once-off activities may be easier to access to coordinate or be less reliant on having services that are accessible to people living in areas of greater socioeconomic disadvantage. However, broader contextual factors such as the COVID-19 pandemic may account for why the relationship between capital plan utilisation and socioeconomic advantage was significant, in contrast to utilisation rates from the year before.

It is worth noting that the variance explained by ISRAD when predicting utilisation rate did not differ substantially when SIL and SDA clients were excluded (SILSDA). Similarly, the relationship between utilisation and socioeconomic advantage remained significant across almost all support class activities – with the exception of capital support in June 2020. The linear relationship between higher utilisation and socioeconomic advantage appeared most strongly when analysing utilisation rates for capacity building budgets.

This finding is significant both for the NDIS and for personalisation programs internationally. It indicates that participants living in lower socio-economic areas (or of low socio-economic status) require additional supports in implementing or using their personalised budgets. In short, it is not enough for government to look at the overall sum of money given to determine if personalisation schemes are functioning effectively and equitable – they need to also look at if that money is being spent. Our analysis does not enable us to examine the causes of this underspend, but it does suggest that more attention needs to be given to this issue. Budget underspend can emerge from a range of issues, including but not limited to:

• The availability of the right or desired service in a local area (including both a complete absence of the service, or long wait lists)
• The NDIS plan and associated budget does not contain the right types and/or amount of supports for the participant’s needs
• The need is periodic or episodic, such as in some mental health based conditions, and is included in the individual funds in the case of a need that has not yet arisen
• The capacity of the NDIS participant has increased and the service is no longer needed
• The capacity of the NDIS participants to contract services is affected by the social determinants to health

In the NDIS, fund underspend and delays in implementing budgets have been a longstanding issue within the scheme as a whole [21]. On an individual participant level there have been media reports of funds with underspend being cut or halved in the subsequent planning year (Morton, 2021). However, until now budget underspend has been presumed to be a “whole of scheme issue”, rather than an issue associated with specific groups (and in particular, an equity issue). This indicates that further investigation through qualitative work is urgently needed in the NDIS to understand why inequitable budget spend is occurring.

While the issue of budget underspend and what is occurring in low socioeconomic areas requires more detailed investigation, there are some shifts to the administrative structures of the NDIS that could be undertaken based on the data presented in this article. The NDIS has a position known as ‘local area coordinators’ [18, 24]. Local area coordinators meet face-to-face with NDIS participants both at the planning stage and, once the budget is approved, to help participants implement these in their budgets and access services. As a result of pressure in the roll out of the scheme, local area coordinators have been pulled away from implementation work to focus on planning work [24, 25]. There have been calls from government reviews, as well as academics studying the scheme, to reinstate the local area coordinator position as one who provides an interface between participants and the service providers, in order to help budget utilisation [24, 26]. The fact that people in low socio-economic areas area struggling to utilise their NDIS budgets puts renewed emphasis on these calls.

Previous research into inequalities in personalisation [6, 10] found that the administrative complexity of such schemes may present problems for equity. They argued that both empirical research, and sociological theories of class, indicate that those in lower socio-economic positions are less likely to have the skills and resources to successfully navigate complex administrative schemes. The findings of this article are consistent with these arguments?Low socio-economic groups may struggle to navigate the implementation stage of their budgets, pointing to the need to provide more targeted supports to these groups. Without effective supports for plan implementation and budget utilisation, the NDIS is positioned to worsen existing social inequalities.

## Limitations

Data availability and transparency are a noted issue when evaluating the impact of the NDIS on social equity (PWDA, 2021). This analysis uses the primary source of publicly available data provided by the NDIA. Providing data as averages for each service district erases variability in both approved budgets and utilisation rates. In this analysis primary explorations establishing linear relationships between ISRAD scores as the independent variable and its relationship to approved budgets and utilisation rates is an early descriptive analysis. However greater data availability is needed to be able to better understand the role of socioeconomic advantage in explaining, in particular, rates of plan utilisation. Current data can account for age groups, but does not provide information on other known influential equity factors such as gender, education level, and cultural heritage.

## Conclusion

Our study found that people living in low socio-economic areas are less likely to successfully utilise their NDIS budgets than those in more affluent areas. This points to the very real possibility that the NDIS, in its current administrative and bureaucratic structure, is entrenching social inequalities. While more in-depth qualitative research is needed into what is occurring for participants in low socioeconomic areas that is leading to poorer budget utilisation, the national quantitative data and findings indicate that more targeted support for budget implementation is needed in lower socio-economic areas.

## Data Availability

The dataset generated and analysed during the current study are available in the Open Science Framework repository: https://osf.io/8dxkn/
